# Quantitative characteristics of clustered DNA damage in irradiated cells by heavy ion beams

**DOI:** 10.1093/jrr/rrt173

**Published:** 2014-03

**Authors:** Hiroaki Terato, Yuka Shimazaki-Tokuyama, Yuko Inoue, Yoshiya Furusawa

**Affiliations:** 1 Saga University, Saga, Japan; 2 National Institute of Radiological Sciences, Chiba, Japan

**Keywords:** heavy ion beam, clustered DNA damage, LET, RBE

## Abstract

Heavy ion beam as typical high linear energy transfer (LET) radiation produces more expanding ionization domain around their tracks than low LET radiation such as X-rays and gamma rays. Thus, heavy ion beam can cause more densely accumulated damage cluster in the target DNA, termed clustered DNA damage. This damage exhibits difficulty for repair and inhibition of DNA replication with its complex structure [ 1]. So, clustered DNA damage is thought to be strongly involved in the biological effectiveness of heavy ion beam. However, a lot of studies have presented no certain correlation between yields of clustered DNA damage and severity of radiation effect. We previously indicated that the yields of clustered DNA damage decreased with increasing LET in the DNA molecules irradiated in test tubes with gamma rays, and carbon and iron ion beams whose showed different LET, respectively [ 2]. In this study, we aimed to reveal correlation between clustered DNA damage and the LET of heavy ion beam in the irradiated cells.

In the experiments, Chinese hamster ovary AA8 cells growing exponentially were irradiated by carbon, silicon, argon and iron ion beams from Heavy Ion Medical Accelerator in Chiba (HIMAC) of the National Institute of Radiological Sciences, Japan. These LETs were 13, 55, 90 and 200 keV/µm, respectively. For comparison, we used gamma rays from ^137^Cs-gamma source, Gammacell 40 (Atomic Energy of Canada Ltd), at Saga University. The irradiated cells were subjected by static-field gel electrophoresis to quantify clustered DNA damage of the genomic DNA. For this analysis, we used Fpg and endonuclease III for clustered DNA damage including oxidative purine and pyrimidine lesions, respectively. We also analysed the corresponding isolated DNA damages by aldehyde reactive probe method [ 3], and the surviving fractions of the irradiated cells in this study.

The electrophoretic results indicated that total yields of clustered DNA damage in the irradiated cells decreased with increasing LET, including the double-strand break (DSB) and the respective clustered base damages (Fig. 1). This result conforms to our previous study with the irradiated DNA molecules [ 2]. The damage kinetics is thought to be mainly derived from two reasons: decreasing fluxes and increasing reaction with reactive oxygen species each other in increase in LET. In the clustered DNA damage induced by each radiation, the most decremental fraction was clustered base damage, but not DSB. The isolated DNA damages decreased with increasing LET like clustered DNA damage in this study (data not shown). These results make us realize the degree of contribution of direct and indirect effects of ionizing radiation. The certain amount of DSB were derived from the direct effect and showed less reactivity to LET. In contrast, oxidative base lesions were mainly generated by indirect effect with reactive oxygen species, which sensitively responded to LET change. We also found seemingly conflicted result of the relationship between LET and RBE (data not shown). We need further study to elucidate act of clustered DNA damage in radiobiological effect with heavy ion beams.

**Fig. 1.**
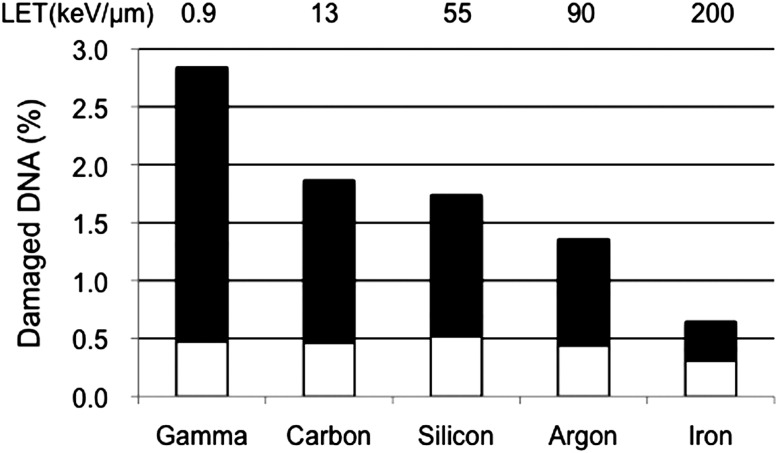
The yields of clustered DNA damages in the cells irradiated with respective ionizing radiations. Each clustered DNA damage consists of DSB (open bar) and clustered base damage (closed bar), and calculated from the strength of released band on electrophoretic gel.

The yields of clustered DNA damages in the cells irradiated with respective ionizing radiations. Each clustered DNA damage consists of DSB (open bar) and clustered base damage (closed bar), and calculated from the strength of released band on electrophoretic gel.

Clinical trial registration number if required: None.
